# Id3 induces an Elk-1–caspase-8-dependent apoptotic pathway in squamous carcinoma cells

**DOI:** 10.1002/cam4.427

**Published:** 2015-02-18

**Authors:** You-Shin Chen, Joseph Aubee, Kyle A DiVito, Hengbo Zhou, Weiyi Zhang, Fen-Pi Chou, Cynthia M Simbulan-Rosenthal, Dean S Rosenthal

**Affiliations:** 1 Department of Biochemistry and Molecular & Cellular Biology, Georgetown UniversityWashington, District of Columbia, 20057; 2 Institute of Biochemistry and Biotechnology, Chung Shan Medical UniversityTaichung, 402, Taiwan; 3 Lombardi Comprehensive Cancer Center, Georgetown UniversityWashington, District of Columbia, 20057

**Keywords:** Apoptosis, caspase-8, Elk-1, HLH, Id3, SCC

## Abstract

Inhibitor of differentiation/DNA-binding (Id) proteins are helix–loop–helix (HLH) transcription factors. The Id protein family (Id1–Id4) mediates tissue homeostasis by regulating cellular processes including differentiation, proliferation, and apoptosis. Ids typically function as dominant negative HLH proteins, which bind other HLH proteins and sequester them away from DNA promoter regions. Previously, we have found that Id3 induced apoptosis in immortalized human keratinocytes upon UVB exposure, consistent with its role as a tumor suppressor. To investigate the role of Id3 in malignant squamous cell carcinoma (SCC) cells (A431), a tetracycline-regulated inducible system was used to induce Id3 in cell culture and mouse xenograft models. We found that upon Id3 induction, there was a decrease in cell number under low serum conditions, as well as in soft agar. Microarray, RT-PCR, immunoblot, siRNA, and inhibitor studies revealed that Id3 induced expression of Elk-1, an E-twenty-six (ETS)-domain transcription factor, inducing procaspase-8 expression and activation. Id3 deletion mutants revealed that 80 C-terminal amino acids, including the HLH, are important for Id3-induced apoptosis. In a mouse xenograft model, Id3 induction decreased tumor size by 30%. Using immunofluorescent analysis, we determined that the tumor size decrease was also mediated through apoptosis. Furthermore, we show that Id3 synergizes with 5-FU and cisplatin therapies for nonmelanoma skin cancer cells. Our studies have shown a molecular mechanism by which Id3 induces apoptosis in SCC, and this information can potentially be used to develop new treatments for SCC patients.

## Introduction

Squamous cell carcinoma (SCC) is one of the most common types of cancer and patients with advanced SCC have a median survival time of only 6–9 months 1. Understanding the molecular mechanism by which SCC tumor cells can be eliminated would therefore benefit a great number of patients.

The inhibitor of differentiation/DNA-binding (Id) family of proteins (Id1–Id4) are dominant negative helix–loop–helix (HLH) transcription factors, which heterodimerize with other HLH proteins, including E proteins and MyoD 2, and inhibit their transcriptional activity by preventing them from binding to DNA promoter regions.

The roles of Id proteins are cell context specific. Ids have been shown to be cooperating oncogenes in many studies, including breast cancer (Id1 and Id3) 3, retinoblastoma (Id2) 4, and melanoma (Id2–Id4) 5. Conversely, Id proteins also promote apoptosis. Overexpression of Id1–Id3 proteins induces apoptosis in primary rat cells in low serum 6, and in B lymphocyte progenitor cells in response to TGF-*β* 7. Id3 also sensitizes immortalized human keratinocytes to X-ray- and UVB-induced apoptosis 8,9. In addition, Id proteins induce apoptosis in tumor cells, including osteosarcoma (Id2) 10 and Burkitt's lymphoma (Id3) 11. Id3 also sensitizes the sarcoma cell line MG-63 to cisplatin 12.

Genetic aberrations of Id3 suggest that it may function as a tumor suppressor. More than 60% of neuroblastoma patients harbor an Id3 deletion in chromosome 1p36 13. In addition, Id3 is identified as a tumor suppressor gene for Burkitt's lymphoma, as inactivating mutations occur in 68% of cases 11,14. Thus, there is a link between the ability of Id3 to induce apoptosis and function as a tumor suppressor gene.

In addition to bHLH, Id proteins have been shown to bind and regulate other transcription factors, including the ternary complex factors (TCF, Elk-1, Elk-4/SAP-1, and Elk-3/SAP-2) 15. TCF proteins are involved in apoptosis. For example, Elk-1 mediates sodium arsenite-induced apoptosis in HaCaT keratinocytes 16. In addition, in response to secreted protein, acidic and rich in cysteine (SPARC) expression, Sp1 and Elk-1 bind to the *caspase-8* promoter and induce apoptosis in MIP101 colorectal cancer cells 17. Elk-1 is also involved in expression of death receptor 5 (DR5) in response to celecoxib in lung cancer cells 18.

Previously, we found that Id3 induced apoptosis in immortalized human keratinocytes 9. To investigate the role of Id3 in malignant SCC cells, a tetracycline (Tet)-inducible system was used to study effects of Id3 in cell culture and mouse xenograft models. This inducible system was chosen to avoid selection of clones that may acquire genetic alteration to tolerate constitutive Id3 expression.

We found that Id3 induction reduced A431 cell numbers in culture under low serum conditions accompanied by an increase in the sub-G1 population. We conclude that this Id3-mediated apoptosis pathway is Elk-1- and caspase-8-dependent for several reasons. First, Id3 induces *elk-1* mRNA and protein as determined by microarray, RT-PCR, and immunoblot analysis. Second, siRNA inhibition of Elk-1 blocks both procaspase-8 and active caspase-8. Third, when A431 cells were treated with caspase-8 and pan-caspase inhibitors, Id3 no longer decreased cell number in low serum or in soft agar assay.

Using Id3 deletion mutants, we found that Id3-induced apoptosis is mediated through its HLH and C-terminal domain (80 amino acids from the C-terminus). Further, Id3 sensitized SCC cells to chemotherapeutic agents including cisplatin and 5-FU (5-fluorouracil). Our studies show that Id3 mediates apoptosis in A431 cells through an Elk-1–caspase-8-dependent pathway. This research can potentially help the development of targeted therapy for SCC patients.

## Materials and Methods

### Cell culture

Human SCC lines A431, SCC4, SCC9, and SCC25 cells were obtained from Georgetown University Tissue Culture Shared Resources. A431 cells were maintained in DMEM/1% penicillin and streptomycin/10% fetal bovine serum (FBS). Tet-approved FBS (Clontech, Mountain View, CA) was used for inducible cell lines. Other SCC cell lines were maintained in DMEM/F-12 50:50/1% penicillin and streptomycin/10% FBS.

### Plasmids

Full-length Id3 and Id3 deletion mutants were cloned into pCDNA4/TO (Invitrogen™, Carlsbad, CA) using *Xho*I and *Xba*I restriction sites. Id3 was subcloned from the pCMS/EGFP-Id3 construct 9. Id3 truncations were cloned using the following primer sequences—ΔN39 (primers 1, 2), ΔC38 (primers 3, 4), ΔN81 (primers 2, 5), and ΔC80 (primers 3, 6):

1. 5′-ACTGCTCGAGGCCGCCACCATGAACATGTTGCTGGACGACATGAACCACTG-3′
2. 5′-ACTGTCTAGATCACTTATCGTCGTCATCCTTGTAATCGTGGCAAAAGCTCCTTTTGTCG-3′
3. 5′-ACTGCTCGAGGCCGCCACCATGAAGGCGCTGAGCCCGG-3′
4. 5′-ACTGTCTAGATCACTTATCGTCGTCATCCTTGTAATCCTGCAGGTCGAGAATGTAGTC-3′
5. 5′-ACTGCTCGAGGCCGCCACCATGGTAGTCCTGGCCGAGC-3′
6. 5′-ACTGTCTAGATCACTTATCGTCGTCATCCTTGTAATCGCTCAGCGGCTCCTCAGC-3′

### Inducible cell lines

A431/Id3 Tet-inducible lines were established by transfecting cells with Tet repressor, pCDNA6/TR (Invitrogen™) using Lipofectamine-LTX (Invitrogen™) followed by selection with blasticidin (7.5 *μ*g/mL). A431/TR cells were then transfected with pCDNA4/TO, pCDNA4/TO-Id3 or pCDNA4/TO-Id3^mutant^ constructs, followed by selection with Zeocin™ (250 *μ*g/mL; Life Technologies, Frederick, MD). Further, pLHCX/GFP and pLHCX/Ds-Red constructs were used to transduce A431/Id3 and A431/Vc cells, respectively, using retroviral packaging cell line ΦNX (Phoenix; ATCC, Manassas, VA) followed by selection with hygromycin (80 *μ*g/mL).

### Growth curves

In six-well plates, 3 × 10^4^ cells were plated per well, and growth curves for ±Tet cells determined by Trypan Blue exclusion assays. For cocultures, 1.5 × 10^4^ green fluorescent protein (GFP) A431/Id3 and Ds-Red A431/Vc cells were mixed together. Five fluorescent images were captured for each well for ±Tet cells and percentages of total area occupied by cells quantified using ImageJ software (National Institutes of Health, Bethesda, MD). The same procedure was employed to determine growth curves with caspase inhibitors (20 *μ*mol/L of Z-IETD-fmk or Z-VAD-fmk).

### Cell cycle analysis

±Tet cells were collected, fixed in ethanol, stained with propidium iodide to determine DNA content, and analyzed by Flow Cytometry (FACStar; BD BioSciences, San Jose, CA).

### Microarray analysis

±Tet cDNA in both A431/Id3 and A431/Vc cells were hybridized to Affymetrix HG-U133A 2.0 gene chips. Gene expression raw data was normalized using dChip software developed by Wong and Lin 19, and analyzed with MetaCore™ software (Thomson Reuters, Washington, DC). Microarray data have been uploaded to Gene Expression Omnibus database (Accession GSE64535).

### siRNA transfection

A431/Id3, SCC4, SCC25, and SCC9 were transfected with 30 pmol of siRNAs (Santa Cruz Biotechnology, Dallas, TX) using RNAi Max (Invitrogen™).

### Tumor xenografts

In athymic nude mice, 1 × 10^6^ GFP-labeled A431/Id3 cells were injected subcutaneously along with 0.5 × 10^6^ Ds-Red-labeled A431/Vc cells. Animals fed with ±Doxycline (Dox) diet (200 mg/kg; Bio-Serv, Flemington, NJ) were monitored for tumor sizes using the Maestro™ In-Vivo Imaging System (CRi, Woburn, MA), as described in the approved Georgetown University IACUC protocol. To monitor xenograft sizes, we use Maestro™ software to track GFP fluorescent signal (peaks at 515 nm), and tumor sizes are measured by the software using an area-defined measurement system.

### Immunoblot analysis

Protein lysates were sonicated and subjected to Tris-glycine sodium dodecyl sulfate polyacrylamide gel electrophoresis (SDS-PAGE) followed by transfer to nitrocellulose membranes. Membranes were then blocked for 1 h with 5% nonfat milk, incubated with primary antibody overnight and secondary antibody for 1 h. The primary antibodies included: anti-Id3 (C-20; Santa Cruz Biotechnology), anti-GAPDH (EMD; Millipore, Billerica, MA), anti-active caspase-3 (Enzo Life Sciences, Inc., Farmingdale, NY), anti-caspase-8 (Thermo Scientific Rockford, IL), and anti-Elk-1 (I-20; Santa Cruz Biotechnology).

### Immunostaining

Paraffin-embedded tumor sections were deparaffinized with xylene followed by antigen retrieval in 6.5 mmol/L sodium citrate at 95°C, pH 6.0. Sections were incubated with antisera specific for active caspase-3 (Asp174; Cell Signaling Technology, Danvers, MA) or Ki-67 (Novus Biologicals, Littleton, CO), overnight at 4°C. Sections were then stained with Alexa 594-conjugated secondary antibodies (Invitrogen™) for 1 h and visualized by fluorescence microscopy. Cell scoring was performed using MetaMorph software (Molecular Devices, Sunnyvale, CA).

### Real Time quantitative Reverse Transcription Polymerase Chain Reaction (qRT-PCR)

One step RT-PCR (Applied Biosystems, Foster City, CA) was performed using 1 *μ*g of total RNA. The two-step qRT-PCR was performed using 100 ng of total RNA. Relative Id3 expression levels were normalized to GAPDH levels using 2^−ΔΔCt^ quantification method.

### Soft agar growth assay

GFP-expressing A431/Id3 cells were pretreated with vehicle (dimethyl sulfoxide), or 20 *μ*mol/L caspase-8 inhibitor (Z-IETD-fmk) or pan-caspase inhibitor (Z-VAD-fmk) overnight. In 250 *μ*L of 0.3% agarose, 10^3^ cells were embedded and added on top of 0.6% agarose base layer 5. Colonies formed in soft agarose were visualized under epifluorescence microscope and counted manually (*N* > 200/group).

### 5-FU and cisplatin treatment

In six-well plates, 3 × 10^4^ cells per well were plated. ±Tet cells were treated with 10 *μ*mol/L of 5-FU (Sigma-Aldrich, St. Louis, MO) or 20 *μ*mol/L of cisplatin (Sigma). Seventy-two hours after drug treatment, cell viability was quantified by Trypan Blue exclusion assay. Mice bearing A431 xenografts were also treated twice with 5-FU (100 mg/kg) and cisplatin (6 mg/kg) by intraperitoneal injection. 5-FU treatments were administrated on days 11 and 18, and cisplatin on days 16 and 23. Tumor sizes were monitored using the CRi Maestro™ In-Vivo Imaging System.

### Statistical analysis

Student's *t-*tests were performed and *P *≤ 0.05 are considered statistically significant (represented by one asterisk). *P *≤ 0.01 or 0.001 are represented by two or three asterisks, respectively. Error bars show mean ± SD unless otherwise specified. To test whether Id3 was synergistic with cisplatin (cis-diamminedichloroplatinum(II); CDDP) or 5-FU, CompuSyn software (ComboSyn, Inc., Paramus, NJ) was used to calculate the combination index (CI) based on the Chou–Talalay drug synergy quantification model 20, using Id3/CDDP or Id3/5-FU ratios ranging from 0.01 to 2000.

## Results

### Establishment of A431/Id3 and A431/Vc cell lines

**Figure 1:**
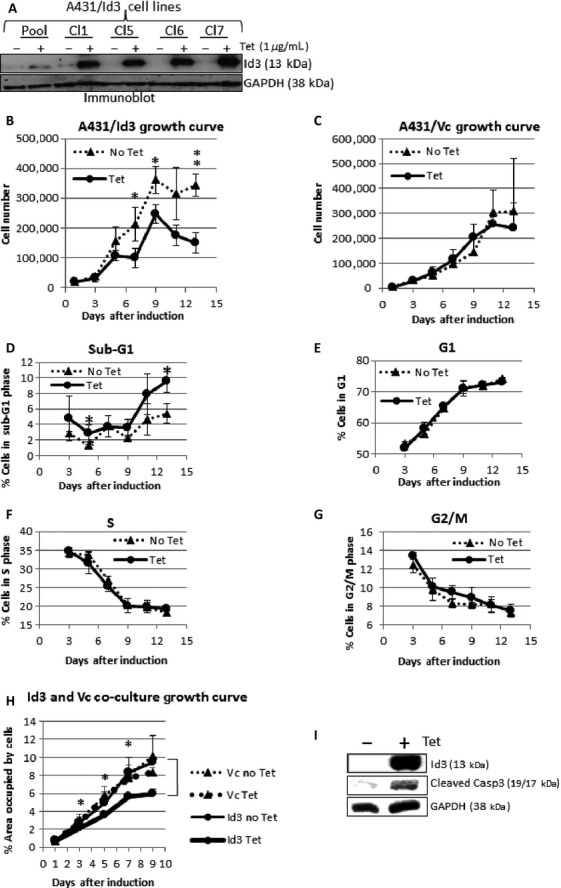
(A) Immunoblot result showing inducibility of Id3 in A431 stable cell lines (pooled clones and four representative clones) by tetracycline (1 *μ*g/mL) for 24 h. GAPDH was used as a loading control. Clone 5 (Cl5) was chosen to proceed with experiments described. (B) A431/Id3 cells were cultured in low serum (0.1% FBS) and viable cell numbers were recorded for 13 days for both uninduced (no Tet) and induced (Tet) groups. (C) A growth curve for A431/Vc cells was determined under the same conditions as A431/Id3 cells. (D–G) A431/Id3 cells were cultured in 0.1% serum and cell cycle analysis performed to determine percentages of cells in phases of the cell cycle. (H) GFP-expressing A431/Id3 and Ds-Red-expressing A431/Vc were cocultured in 0.1% serum. Growth curves were determined and statistical analyses performed to determine any significant differences in growth. (I) Immunoblot showing Id3 and caspase-3 cleavage products for A431/Id3 cells. FBS, fetal bovine serum. *P* values ≤ 0.05 are considered statistically significant and represented by asterisks.

To investigate the effects of Id3 in malignant SCC cells, a Tet-inducible system was used to induce Id3 expression. First, stable cell lines were verified for Id3 protein induction by Tet (Fig. 1A) and a robust induction of Id3 mRNA (seven- to eightfold) was confirmed by semiquantitative reverse transcriptase-mediated PCR (RT-PCR) as well as by qRT-PCR (Fig. S1). A Tet-induction time course was performed in A431/Id3 clone 5 and A431 vector control (A431/Vc) cell lines. The induction of Id3 in the A431/Id3 cl5 cell line can be seen as early as 2 h after addition of Tet, and a decrease observed after 16 h. There was no detectable Id3 protein in A431/Vc cells throughout the time period tested (Fig. S2). Normalized Id3 protein expression levels relative to GAPDH loading control are shown in Figure S2.

### Cells with induced Id3 expression showed decreased cell numbers in low serum

Since Id proteins are associated with cell cycle progression and survival 6,21, we first determined whether induction of Id3 alters cell growth. In normal serum, no differences were observed between ±Tet cells. A431/Id3 cells were then cultured in low serum (0.1% FBS) conditions and we observed that Id3 induction resulted in significantly fewer viable cells compared to uninduced cells (Fig. 1B). We then examined if the decrease in cell number was concomitant with changes in the cell cycle. We observed an increase in the sub-G1 cell population in Id3-induced cells (Fig. 1D) but no changes in S phase (Fig. 1F) or in other phases of the cell cycle (Fig. 1E and G). The growth curve of A431 Vc cells was also assayed in the same manner and no differences in cell number were observed, indicating that tetracycline did not alter cell cycle or cell death (Fig. 1C).

We next cocultured GFP-expressing A431/Id3 and Ds-Red-expressing A431/Vc cells and examined cell growth ±Tet in low serum conditions (Materials and Methods). Student's *t*-tests revealed that coculture with induced A431/Id3 cells did not alter proliferation of A431/Vc cells (Fig. 1H), indicating that changes in cell numbers were not due to cell–cell communication or secreted factors.

To identify the form of cell death induced by Id3, we determined whether caspase-3, the converging point for most apoptotic pathways, was proteolytically processed from procaspase-3 into its smaller, active form. Upon Id3 induction, active caspase-3 subunits were clearly observed (Fig. 1I). This indicates that Id3 induction is sufficient to induce apoptosis in A431 cells by a pathway most likely involving caspase-3.

### Microarray analysis revealed Elk-1 as a downstream target of Id3

**Figure 2:**
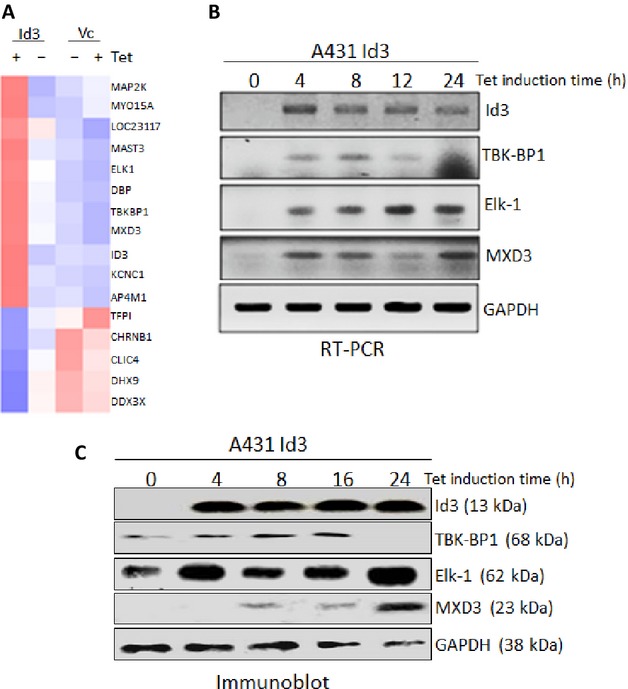
(A) Heat map showing 16 genes significantly up- or downregulated by Id3 by at least 1.5-fold. (B) RT-PCR was performed to verify genes that were shown to be upregulated by Id3 in microarray analysis. (C) Immunoblot analysis of proteins corresponding to genes upregulated by Id3.

To investigate the molecular pathways by which Id3 mediates caspase-3 activation and apoptosis in A431 cells, microarray analysis was performed. A431/Id3 and A431/Vc ±Tet cells were subjected to microarray analysis. Id3 altered expression levels of 16 genes greater than 1.5-fold, when induced A431/Id3 was compared with the other three groups (Fig. 2A). We verified expression levels of candidate genes and showed that TBK-BP1, Elk-1, and MXD3 were upregulated at the mRNA (Fig. 2B) and protein (Fig. 2C) levels by Id3.

### Id3 induced apoptosis through an Elk-1–caspase-8-dependent pathway

Of candidate genes, Elk-1 was the only gene that physically interacts with Id proteins 15. Furthermore, Sp1 and Elk-1 bind to the caspase-8 promoter and induce apoptosis in colorectal cancer cells in response to SPARC treatment 17. We therefore investigated the potential involvement of Elk-1 and caspase-8 in Id3-induced apoptosis in A431 cells.

**Figure 3:**
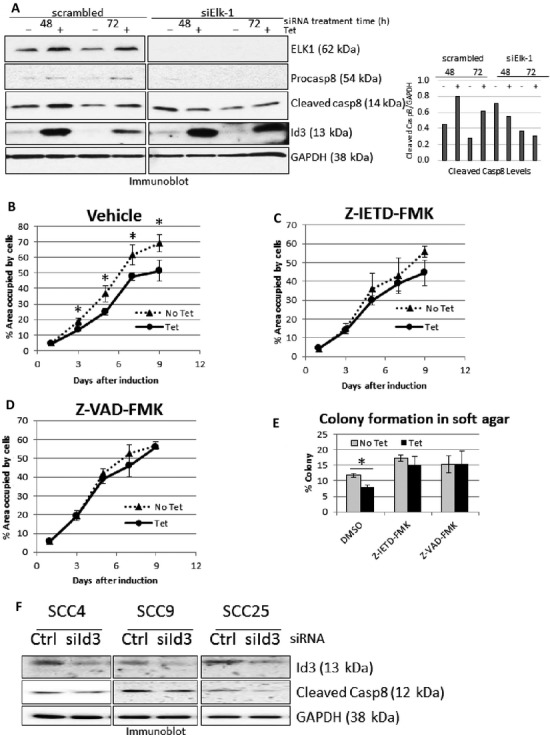
(A) A431/Id3 cells in 0.1% FBS were treated with either scrambled or Elk-1 siRNA (siElk-1) for 48 or 72 h and assayed for protein levels. Densitometric quantification of immunoblot showing relative cleaved caspase-8 levels compared to those of GAPDH. (B–D) GFP-expressing A431 Id3 cells were treated with vehicle (B, DMSO), caspase-8 inhibitor (C) or pan-caspase inhibitor (D) in 0.1% FBS. Cell growth was assayed. (E) GFP-expressing A431/Id3 cells treated with either vehicle (DMSO), caspase-8, or pan-caspase inhibitor were grown in agarose and assayed for colony formation after 4 days. (F) SCC4, SCC9, and SCC25 cells were treated with scrambled or Id3 siRNA for 24 h, and assayed for Id3 and caspase-8 levels by immunoblot analysis. SCC, squamous cell carcinoma; FBS, fetal bovine serum. *P* values ≤ 0.05 are considered statistically significant and represented by asterisks.

Upon Id3 induction, A431/Id3 cells showed elevated levels of Elk-1, procaspase-8, and active caspase-8 proteins (Fig. 3A). When cells were transfected with Elk-1 siRNA, Id3 induction of Elk-1, procaspase-8, and active caspase-8 were all suppressed (Fig. 3A). This suggests an apoptotic pathway leading from Id3 to Elk1 to procaspase-8 induction and activation in A431 cells.

### Caspase inhibitors abolished Id3-induced decreases in cell numbers in low serum, and colony formation in soft agar

To further investigate the involvement of Id3 in apoptosis, we treated cells with caspase-8 and pan-caspase inhibitors. As before, Tet-induction of Id3 reduced cell numbers in the presence of vehicle (Fig. 3B), while the caspase inhibitors attenuated effect of Id3 on cell number decrease in low serum (Fig. 3C and D). This indicates that Id3-induced decrease in cell number under low serum is caspase dependent.

We next investigated the ability of A431/Id3 cells to form colonies in agarose. We observed a significant decrease in cell colonies when Id3 was induced (Fig. 3E). However, in the presence of caspase inhibitors, the decrease in colony formation due to Id3 induction was eliminated (Fig. 3E), indicating that Id3 inhibition of soft agar colonies is also caspase dependent.

To further investigate the induction of caspase-8 activation by Id3, we reduced endogenous Id3 levels using siRNA in SCC4, SCC9, and SCC25 cells. Knocking down Id3 decreased levels of active caspase-8 in these other SCC cells (Fig. 3F) when quantified by densitometry: SCC25 (74%) > SCC9 (38%) > SCC4 (17%). Our results suggest that endogenous Id3 induces spontaneous apoptosis via a caspase-8-dependent pathway in other SCC lines.

### Id3-induced apoptosis is C-terminus and HLH dependent

**Figure 4:**
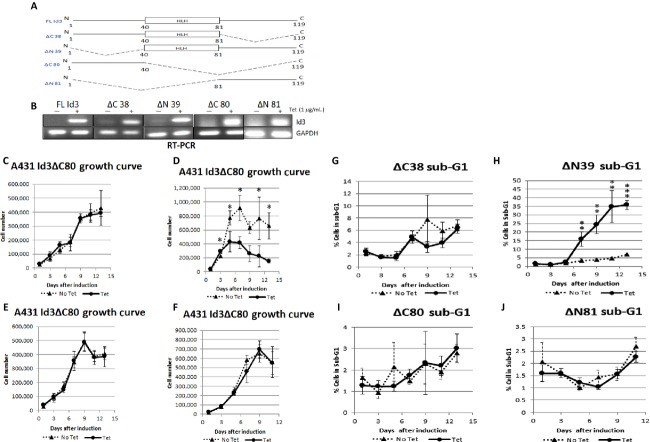
(A) Schematic representation of Id3 deletions of the C-terminus (ΔC38), N-terminus (ΔN39), C+HLH (ΔC80) domain, or N+HLH (ΔN81) domain. (B) Induction of Id3 mutants by Tet (1 *μ*g/mL for 24 h) were verified. (C–F) Growth curves for Id3-mutant cell lines. (G–J) Cell cycle analyses performed for Id3-mutant cell lines. HLH, helix–loop–helix. *P* values ≤ 0.05 are considered statistically significant and represented by asterisks.

To investigate the domains of Id3 that are responsible for apoptosis, four inducible cell lines expressing Id3 deletion mutants were created and verified (Fig. 4A–B). We observed that deleting the N-terminus of Id3 (ΔN39) did not abolish Id3-induced apoptosis (Fig. 4D). Other Id3 mutants all lost their ability to reduce cell numbers (Fig. 4C, E–F). Further, Id3 ΔN39 showed a drastic increase in sub-G1 population (Fig. 4H), while other mutants showed no significant difference in sub-G1 population (Fig. 4G, I–J). These results suggest that the HLH and C-terminal domains of Id3 gene are important for induction of apoptosis in A431 cells.

### Id3 induction decreased xenograft sizes in vivo

**Figure 5:**
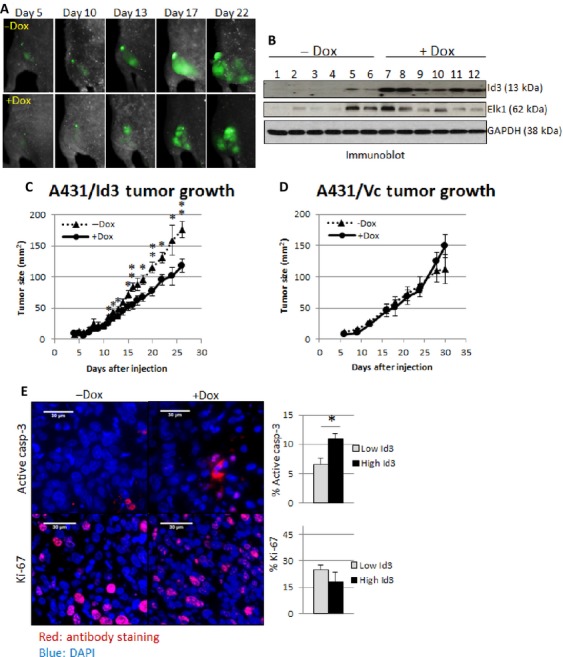
(A) Mouse xenografts formed from GFP-expressing A431/Id3 cells imaged in vivo. (B) Id3 and Elk-1 protein levels were examined in A431/Id3 tumor lysates. (C,D) Xenograft sizes from GFP-expressing A431/Id3 cells and Ds-Red-expressing A431/Vc cells for both uninduced (−Dox) and induced (+Dox) groups were imaged in vivo. Results are expressed as mean ± SE (A431/Id3, −Dox: *N* = 10; +Dox: *N* = 14. A431/Vc, −Dox: *N* = 5; +Dox: *N* = 5). (E) Tumor sections from A431/Id3 xenografts were assayed for levels of active caspase-3 (top row) and Ki-67 (bottom row) by immunofluorescent staining in ±Dox groups. DAPI stains cell nuclei. Percentages of cells stained for both markers are shown in bar graphs on right (low Id3: *N* = 6, high Id3: *N* = 3). *P* values ≤ 0.05 are considered statistically significant and represented by asterisks.

To investigate the ability of Id3 to suppress tumor growth in vivo, we injected A431 cells subcutaneously into mice and tracked tumor growth via in vivo imaging of GFP-expressing cells (Materials and Methods; Fig. 5A). Induction of Id3 in vivo was achieved through doxycycline (Dox)-containing feed (Fig. 5B). We found that Id3 significantly reduces tumor size (≈33%, *P* = 0.002; Fig. 5C). When A431/Vc were injected into nude mice, no differences were observed in the sizes of ±Dox tumors (Fig. 5D). Further, immunoblot analysis showed that Elk-1 is induced in tumors with high levels of Id3 induced by Dox (Fig. 5B).

Immunofluorescent staining was then performed to investigate if reduced Id3 xenograft sizes are due to decreased proliferation or increased apoptosis. We observed a 50% increase in active caspase-3 levels in tumors expressing high levels of Id3 (*P* = 0.01; Fig. 5E). On the other hand, expression of the proliferation marker Ki-67 was unaltered (Fig. 5E). Our data suggest that Id3 decreases xenograft sizes via apoptosis.

### Id3 sensitizes SCC cells to chemotherapeutic drugs

**Figure 6:**
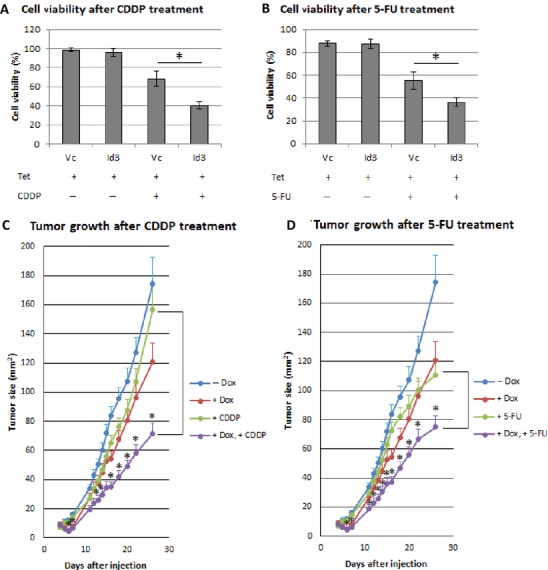
(A–B) Cell viabilities were determined for Tet-induced A431/Id3 and A431/Vc cells treated with CDDP or 5-FU for 3 days. (C–D) A431/Id3 mouse xenografts were fed with control diet or with Dox to induce Id3. Dox-induced or -uninduced mice were then left untreated, or treated with CDDP (C), or 5-FU (D). Tumor sizes were determined as described in Materials and Methods. Results are expressed as mean ± SE (*N* = 6 for each group). Asterisks represent statistically significant differences in tumor sizes after chemotherapy in the presence or absence of Dox-induced Id3. *P* values ≤ 0.05 are considered statistically significant and represented by asterisks.

Id3 has been shown to mediate cisplatin-induced apoptosis in sarcoma cells 12. We therefore investigated the effect of Id3 on cell death in A431 cells in response to 5-FU and CDDP. For both drugs, the CI values for 90% and 50% population death are all less than 1, which is an indication of a synergistic effect (Table1). Thus, Id3 sensitizes A431 cells to CDDP and 5-FU treatments (Fig. 6A–B).

**Table 1:** CI values for Id3 + CDDP and Id3 + 5-FU combination treatments

Id3/CDDP ratio	CI for 90% death	CI for 50% death	Id3/5-FU ratio	CI for 90% death	CI for 50% death
1/2000	1.20E-4	0.00279	1/1000	Infinity	Infinity
1/20	1.22E-04	0.00278	1/10	2.99E-7	5.11E-4
1/2	1.39E-04	0.0027	1	4.36E-7	4.69E-4
1	1.54E-04	0.00263	2	5.64E-7	4.42E-4
5/2	1.88E-4	0.00252	5	8.74E-7	3.99E-4
10	2.84E-4	0.00229	20	1.93E-6	3.32E-4
100	6.28E-4	0.00192	200	8.03E-6	2.39E-4
1000	0.00143	0.00159	2000	8.00E-6	2.39E-4

5-FU, 5-fluorouracil.

We further investigated the efficacy of CDDP or 5-FU in the presence or absence of Id3 in mouse xenografts derived from A431 cells. Our data indicate that CDDP or 5-FU alone decrease xenograft sizes by 10% and 35%, respectively, on the last measured day of growth (day 26). However, in Dox-induced Id3 tumors, CDDP or 5-FU decreases xenograft sizes by 55% (Fig. 6C–D). These results suggest that Id3 levels may be a predictor of drug response or that Id3 itself could serve as a therapeutic target.

## Discussion

We demonstrate that Id3 induced apoptosis in malignant A431 SCC cells in culture and in vivo. In cell culture, we show that Id3 reduced cell numbers in low serum and in soft agar, accompanied by an increase in sub-G1 population. In the mouse xenograft model, we show that Id3 significantly decreases tumor size by >30% via apoptosis. Furthermore, we have shown that Id3 induced Elk-1 in vitro and in vivo. This signaling cascade results in the proteolytic activation of caspases-8 and -3, leading to cell death. As revealed by our Id3 mutant analyses, this pathway is likely dependent on the C-terminal and HLH domains of Id3 (Fig. 4C–F).

The mechanism by which Id3 modulates apoptosis in malignant keratinocytes appears to be different from that of nontransformed keratinocytes. In premalignant cells, Id3 induces *bax* expression at the promoter level 9,22. On the other hand, ectopic expression of Id3 alone did not elevate Bax protein levels in A431 cells. However, Id3 does enhance Bax protein in response to UVB exposure (480 J/m^2^, data not shown).

Deletion of N-terminal 39 amino acids of Id3 renders it a more potent apoptosis inducer (Fig. 4D and H). The mechanism remains to be investigated, although this might be due to the loss of the Cdk2 S5 phosphorylation site. Phosphorylation of Id3 by CyclinA/E-Cdk2 complexes abolishes interaction between Id and TCF proteins 23. Deleting this phosphorylation site from Id3 protein may allow Id3 to escape Cdk2 regulation, and thus interact with Elk-1 with greater affinity.

Id3 siRNA reduced active caspase-8 levels in SCC4, SCC9, and SCC25, which all harbor p53 mutations in their DNA-binding domains. SCC4 and SCC25 have one p53 allele each 24, which is a p53^P151S^ gain-of-function (GOF; SCC4) 25,26, and a p53^R209fs^ 2-bp frameshift (SCC25) 24. SCC9 has a p53^274fs^ 32-bp frameshift. SCC4 overexpresses the GOF p53, while p53 is undetectable in SCC9 and SCC25 24,27–29. The loss of p53 expression did not prevent Id3 siRNA-mediated reduction in caspase-8 activation, especially in SCC25 cells, suggesting that Id3-mediated activation of caspase-8 is p53-independent (Fig. 3F). A431 also has only one p53 allele harboring a GOF p53^R273H^ missense mutation in the DNA-binding domain 30, which downregulates procaspase-3 and increases resistance to chemotherapeutics A431 31. In contrast to p53^R273H^, Id3 induces apoptosis in A431 cells and sensitizes these cells to cisplatin and 5-FU, which is also consistent with p53-independent apoptosis.

We have demonstrated that Id3 sensitizes A431 cells to cisplatin and 5-FU. Id3 has also been shown to sensitize sarcoma cells to cisplatin 12. However, the Id1/Id3 aptamer (Id1/3-PA7) has been shown to induce cell cycle arrest and apoptosis in breast cancer cells 32. Further, Id3 levels in cancer have been shown to be study- and/or cell type-specific. Analysis of the Oncomine® database revealed that Id3 was significantly upregulated in 83 studies and downregulated in 84 studies in which cancers were compared to normal samples (threshold *P *= 0.05, fold change = 1.5, accessed date 18 June 2014). Thus, employing Id3 as a therapeutic target requires careful evaluation, and context-specific effects should be used to inform design of therapeutics when making decisions to treat cancer patients.

## Supporting Information

**Figure S1.** A431/Id3 clone 5 cells were induced by Tet (1 *μ*g/mL) and assayed for Id3 RNA expression by RT-PCR as described in sec 2. GAPDH served as a loading control. qRT-PCR (right) shows Id3 mRNA levels at 0, 4, 8, 12, and 24 h after Tet-induction. Relative Id3 levels were normalized to GAPDH levels in each time point using Ct values. **Figure S2.** (Top): Id3 was detected by immunoblot analysis in A431/Id3 clone 5 and A431/Vc cell lines 0, 2, 4, 8, 16, or 24 h after addition of Tet. Tet was then removed after 24 h. (Bottom): Densitometric quantification of immunoblot showing relative Id3 expression levels compared to GAPDH.
