# Supplementary figures and images for: Id3 induces an Elk-1–caspase-8-dependent apoptotic pathway in squamous carcinoma cells
Source: Cancer Med. 2015 Feb 18;4(6):914–24. doi: 10.1002/cam4.427 (PMC4472214; doi:10.1002/cam4.427)

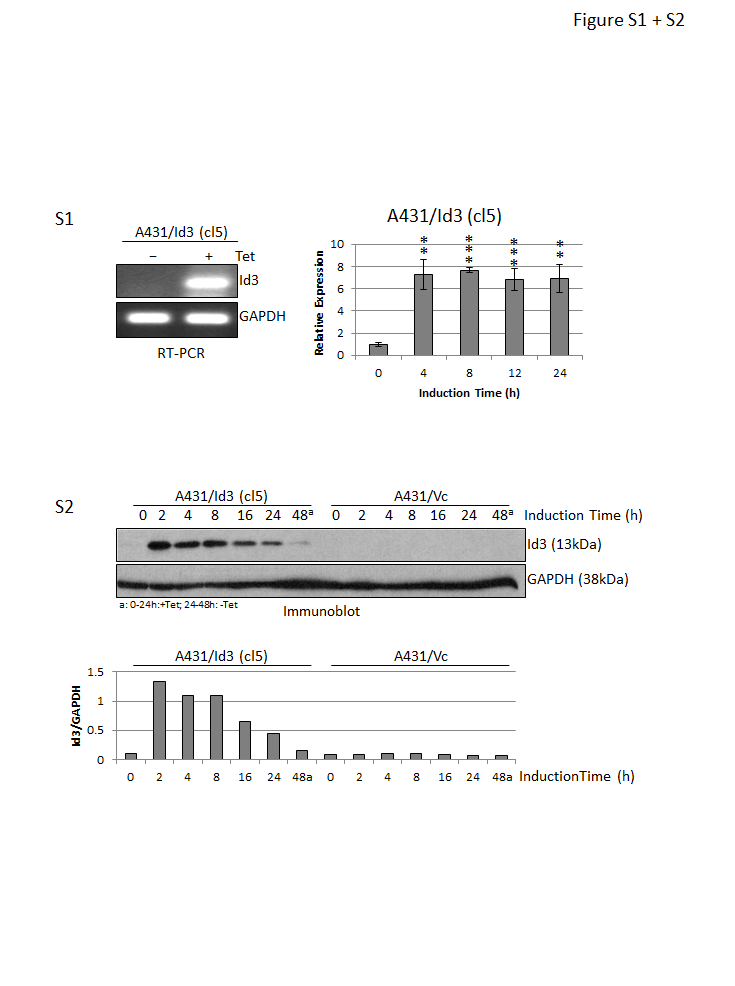

Supplement: Supplementary file 1 [file cam40004-0914-sd1.tif]
